# Maspin as a Prognostic Marker for Early Stage Colorectal Cancer With Microsatellite Instability

**DOI:** 10.3389/fonc.2020.00945

**Published:** 2020-06-10

**Authors:** Atsushi Tanaka, Julia Y. Wang, Jinru Shia, Yihua Zhou, Makiko Ogawa, Ronald C. Hendrickson, David S. Klimstra, Michael H. A. Roehrl

**Affiliations:** ^1^Department of Pathology, Memorial Sloan Kettering Cancer Center, New York, NY, United States; ^2^Department of Pathology, Graduate School of Medicine, University of Tokyo, Tokyo, Japan; ^3^Human Oncology and Pathogenesis Program, Memorial Sloan Kettering Cancer Center, New York, NY, United States; ^4^Curandis, New York, NY, United States; ^5^ICU Department, Second Affiliated Hospital of Nanchang University, Nanchang, China; ^6^Memorial Sloan Kettering Cancer Center, Sloan Kettering Institute, New York, NY, United States

**Keywords:** maspin, serpin B5, colorectal cancer, biomarker, prognosis, pathology

## Abstract

Colorectal cancers are among the most common cancers and a leading cause of cancer death. In our pursuit to discover molecular markers for better characterization and precision theranostics of these cancers, we first conducted global deep proteome analyses and identified maspin (serpin B5, peptidase inhibitor 5) as an upregulated protein in tumor tissue. We then validated its expression in a large cohort of 743 patients with colorectal cancers of all stages and found that both cytoplasmic and nuclear expression varied widely between different patients. Comparison with clinicopathological features revealed that maspin expression levels correlate significantly only with mismatch repair (MMR) status but not with other features. To elucidate the prognostic significance of maspin, we analyzed two outcome-annotated cohorts, one of 572 early stage cancer patients and another of 93 late stage cancer patients. Kaplan–Meier survival, univariate, and multivariate analyses revealed that maspin overexpression predicts longer overall and disease-free survival for early stage microsatellite instability (MSI) subtype colorectal cancer, but there is no correlation with survival for patients with early stage cancer of the microsatellite stability (MSS) subtype or late stage cancer. Our study identifies maspin expression as an independent prognostic marker for risk stratification of early stage MSI subtype colorectal cancer and may provide guidance for improved therapeutic management.

## Introduction

Colorectal cancer (CRC) is the third most common cancer and the third leading cause of cancer death. Risk assessment of early stage cancer is particularly critical because it determines whether adjuvant chemotherapy or other targeted therapies would be beneficial in addition to surgical resection. Because of a lack of reliable prognostic molecular biomarkers, risk assessment of early stage CRC is currently challenging. Morphological features such as poorly differentiated histology, bowel obstruction, localized perforation, and positive margins are often used as prognostic factors (1–3). Molecular biomarkers with more precise prognostic value, preferably with an underlying functional pathophysiologic rationale, are in demand, as such markers would enable better risk stratification of early stage CRC after surgery and more accurate selection of high-risk patients for adjuvant therapy, while avoiding overtreatment in low-risk patients.

Aside from chemotherapies that treat cancer cells non-discriminately, personalized cancer care has been moving toward more precise, molecularly targeted drugs that intervene in specific biological pathways. Immune checkpoint inhibitors have emerged as a standard treatment for CRC, particularly for the subtype with microsatellite instability (MSI). Although MSI has been recognized as a marker for positive response to immune checkpoint inhibitors, additional markers to subclassify MSI cancers are needed as MSI patients clearly do not uniformly respond to the same therapy (4, 5). Despite the great advances in genome-based cancer classifications, it should also be stressed that all current therapeutic targets are protein-based. Hence, proteomic research into new protein markers with prognostic or predictive value is an important and promising task.

In our quest for protein signature markers of colorectal cancer, we initially carried out global non-biased proteomic profiling and identified the overexpression of maspin protein in CRC tissues. Maspin (mammary serine protease inhibitor), also named serpin B5 or peptidase inhibitor 5, is a member of the serpin superfamily. Maspin is expressed in the skin, prostate, testis, intestine, tongue, lung, and the thymus. The primary function of most members of this family is to regulate the breakdown of proteins by inhibiting the catalytic activity of proteases. Through this mechanism of action, serpins regulate a number of cellular processes including phagocytosis, coagulation, and fibrinolysis. According to the Human Protein Atlas database (6), almost all human tissues, including normal colonic mucosa, express some level of maspin. Immunohistochemical and immunofluorescence analyses of maspin show both cytoplasmic and nuclear localization of the protein. However, maspin's biological function or prognostic relevance in colorectal cancer have remained unclear. In addition, we decided to examine maspin more closely based on our recent discovery of serpins (including maspin) as autoantibody-inducing autoantigens in CRC and renal tissue using two different experimental approaches (7, 8). Our repeated identification of maspin as a unique biomarker by orthogonal approaches affirmed our interest in further examining this protein's expression and prognostic value in a large cohort of 743 CRC patients.

## Materials and Methods

### Clinical Specimens and Pathological Data

Adenocarcinoma tissues and normal colonic mucosa were obtained from the Precision Pathology Biobanking Center of Memorial Sloan Kettering Cancer Center. The study was approved by the Institutional Review Board (IRB) of Memorial Sloan Kettering Cancer Center. Data were acquired retrospectively and in an anonymized manner such that patient consent was not required as determined by the IRB. Clinical data, including patient demographics, treatment history, recurrence status, and MMR status, were retrieved from medical records. Histologic type and other pathological parameters were extracted from diagnostic pathology reports, and tumor content ratios for all samples were checked by gastrointestinal subspecialty pathologists.

### Fresh Frozen Tissue Selection

For the initial proteomic discovery of protein biomarkers, we selected 15 CRC cases with tissue sample criteria of high tumor content (>50%), minimal gross and microscopic necrosis (<5%), and low blood contamination (<5%). Matched pairs of frozen tumor tissue and benign colonic mucosa harvested away from the cancer (carefully stripped without muscularis propria) were retrieved from the vapor phase liquid nitrogen repository. Two gastrointestinal subspecialty pathologists verified the diagnoses and quality of all tissues.

### Tissue Proteome Extraction and Digestion

Five-milligram aliquots of frozen tissue were thawed on ice and lysed with 200 μl lysis buffer containing 8 M urea, 0.1 M ammonium bicarbonate, phosphatase inhibitors 2 and 3 (Sigma), and protease inhibitors (Roche). The tissue mixture was homogenized with 12 1-min cycles of sonication at 120 W power (FB120, Fisher Scientific) and intermittent cooling. After centrifugation at 14,000 g for 30 min at 4°C, the supernatant containing all soluble proteins was collected. The protein concentration was determined by a BCA assay (Pierce), and extracted proteomes were stored at −80°C until further analysis.

### Protein Digestion

Aliquots of 50 μg of the extracted proteomes were reduced with 5 mM dithiothreitol at 56°C for 30 min and alkylated with 11 mM iodoacetamide at room temperature for 30 min in the dark. They were then digested with trypsin and Lys-C (0.2 μg/μl, both from Promega) at 1:50 (w/w) at 37°C for 12 h. The digestion was stopped by the addition of trifluoroacetic acid to a final concentration of 1%. The mixture was centrifuged at 14,000 g for 10 min at room temperature. The clear supernatant was collected and desalted on a lab-made C_18_ StageTip. Desalted peptides were dried in a SpeedVac vacuum concentrator and re-dissolved in 10–15 μl of 3% acetonitrile/0.1% formic acid and stored at −20°C.

### Proteome Sequencing by Mass Spectrometry

Approximately 1 μg of desalted peptides was injected into a 50-cm C_18_ capillary column mounted to an Easy-nLC 1200 system coupled to an Orbitrap Fusion Lumos mass spectrometer (Thermo Scientific). Peptides were eluted over a 200-min gradient in 2–35% buffer B [0.1% (v/v) formic acid, 99.9% (v/v) acetonitrile], and buffer A [0.1% (v/v) formic acid, 99.9% (v/v) HPLC-grade water] at a flow rate of 300 nl/min. MS data were acquired with an automatic switch between a full scan and 10 data-dependent MS/MS scans. MS/MS scans were acquired at a resolution of 15,000 at 200 m/z with an ion target value of 5 × 10^4^, maximum injection time of 100 ms, and dynamic exclusion for 15 s in centroid mode. Label-free protein quantification was carried out with MaxQuant (9, 10). A minimum of 1 peptide was required for protein identification, but 2 peptides were required to calculate a protein level ratio. Significantly up-regulated and down-regulated proteins were identified with Perseus software (11, 12).

### Tissue Microarray Construction

Tissue microarrays (TMAs) were constructed from colorectal cancers according to well-established procedures. For the present study, we prepared two independent TMA cohorts. One was constructed from surgically resected cases of stages I and II only. The second was constructed from surgically resected cases of all stages (I–IV). Case selection criteria except clinical stage were the same for both cohorts. Cases whose resection dates ranged from 1981 to 2010 were used for TMA construction. Clinical stages I and II were grouped as “early stage,” while clinical stages III and IV were grouped as “late stage.” Three tissue cores (2 mm diameter each) were punched out from each donor paraffin block and transferred to TMA blocks using a TMA Grand Master robot (3DHistech). Formalin-fixed paraffin-embedded tissues were cut into 4-μm sections.

### Immunohistochemistry (IHC)

Tissue sections were treated with xylene for paraffin removal, and antigens were retrieved by heat-mediated epitope retrieval. Maspin expression in tissue sections was probed with anti-maspin polyclonal antibodies (HPA019025, Atlas Antibodies) at 1:2,000 dilution. IHC staining was conducted with Leica BOND-MAX automation. IHC results were scored by a semi-quantitative approach. Cytoplasmic staining intensity of individual tumor cells was determined and assigned intensities of 0, 1, 2, or 3. The total weighted IHC score (IHC *H*-score) of a sample slide was calculated by multiplying the expression intensity of individual tumor areas (score, 0–3) by their relative contribution (0–100%) to total tumor area and adding these to yield a total weighted sum. The IHC *H*-scores thus have a theoretical range of 0–300. We also assessed nuclear staining as positive if 10% or more tumor cells showed nuclear staining. Assessment of all tissue samples was independently performed by two pathologists without any clinical information. In cases of discrepancies in immunohistochemical assessment between the two pathologists, the cases were reviewed by them together and a consensus score was determined.

### Statistical Analyses

Categorical variables were compared using Fisher's exact test. Numerical values were analyzed by the Mann–Whitney *U*-test. Survival analyses were performed using the Kaplan–Meier method and compared by a log-rank test. Multivariate analyses of prognostic factors were performed with logistic regression models by using factors that showed significant univariate differences (*p* < 0.05). Statistical analyses were performed with the JMP Pro 14 software (SAS). All statistical analyses were considered significant with *p* < 0.05.

## Results

### Maspin Expression in Colorectal Cancer by Global Proteomic Profiling

**Figure 1:**
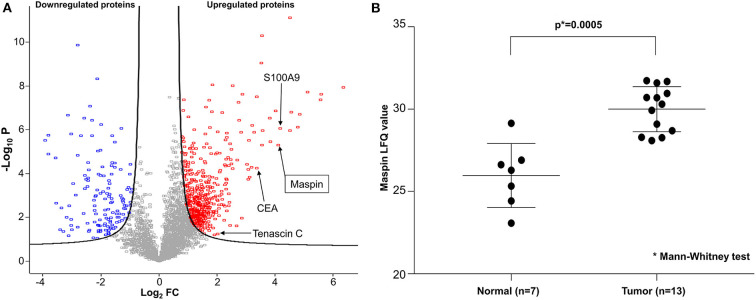
Deep proteomic analysis of CRC by mass spectrometry. **(A)** Volcano plot of quantified protein changes from 15 patients. The curved solid line shows the FDR (false discovery rate) horizon of 0.05. The horizontal axis indicates log_2_-fold change (FC) of protein abundance (cancer relative to matched benign mucosa). The vertical axis shows –log_10_ of the *t*-test *p*-value. Up-regulated proteins are shown in red and down-regulated proteins are shown in blue. Maspin is highlighted by an arrow. **(B)** Statistical comparison of maspin protein levels detected by mass spectrometric label-free quantitation (LFQ).

To discover potential biomarkers for CRC, our first goal was to identify proteins that are differentially expressed in tumor tissues, particularly those that are overexpressed in tumors relative to benign colonic mucosa. For optimal results, we selected cancer tissue samples that had high tumor content, minimal necrosis, and minimal blood contamination. Proteomes were extracted from each tissue, digested to peptides, and sequenced by Fourier transform mass spectrometry. From the proteomic profiling of 15 pairs of adenocarcinoma and matched normal colonic mucosa, 6,158 individual proteins were identified and quantified, and 3,238 proteins were found to be shared by 50% or more of samples. A total of 622 proteins were found to be differentially expressed, with 486 over- and 136 under-expressed in CRC relative to matched benign mucosa (Figure 1A). Reassuringly, several known CRC biomarkers, such as CEA, S100A9, and tenascin C (13–15), were among those overexpressed in the tumor tissues in our findings, validating our experimental approach. Maspin was the 11th most upregulated protein in colorectal tumor tissues with a 17.2-fold protein abundance change relative to normal colonic mucosa (Figure 1A and Supplementary Table 1). Label-free quantification (LFQ) values of maspin were significantly higher in cancer than those in benign colonic mucosa (Figure 1B).

### Maspin Protein Expression Validation

**Figure 2:**
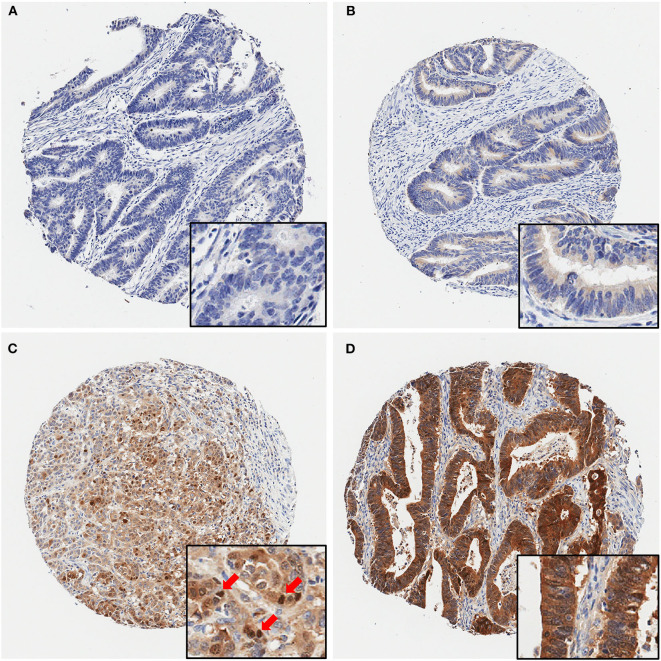
Representative immunohistochemical maspin protein stains showing four different TMA cores. **(A)** Negative (no protein expression), **(B)** weakly positive, **(C)** moderately positive (with several positive nuclei, red arrows), **(D)** strongly positive staining. Original magnifications: 40× (insets, 400×).

Next, we examined maspin expression in CRC by immunohistochemistry in independent clinical cohorts of 743 patients, comprising 628 cases of early stage and 115 cases of late stage CRCs. As shown in Figure 2, maspin expression levels in tissue varied greatly between patients, ranging from negative to strongly positive. Maspin is mostly found in the cytoplasm of cancer cells (Figures 2B–D with inserts). Some cases also showed strong nuclear expression (Figure 2C with insert). Stromal and inflammatory cells were essentially negative for maspin.

**Figure 3:**
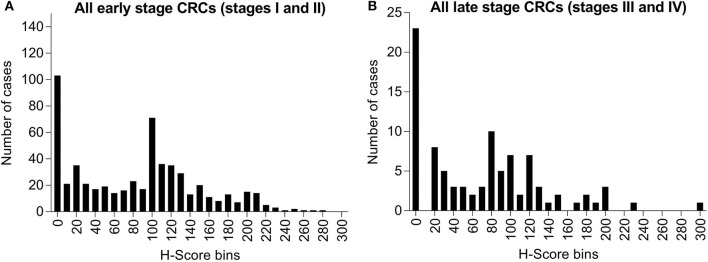
Bar graph of maspin protein expression distribution derived from IHC staining of 743 CRCs. **(A)** Six-hundred and twenty-eight cases of early stage CRC (stages I or II) and **(B)** One-hundred and fifteen cases of late stage CRC (stages III or IV). The horizontal axis indicates *H*-score bins (bin width, 10). The vertical axis indicates the absolute case count for each *H*-score bin.

Among these specimens, 19.4% (122/628) of early stage cases (stages I and II) and 20.0% (23/115) of late stage cases (stages III and IV) had no detectable level of maspin (Figure 3). Within the total IHC *H*-score range of 0–300, early stage cases had mean and median *H*-scores of 82.6 and 90.0, respectively, and late stage cases had mean and median *H*-scores of 70.6 and 65.0, respectively. The overall expression distributions of maspin in early and late stage colorectal cancer tissue were very similar, suggesting that stage progression did not affect maspin expression (Figure 3). The finding that maspin overexpression occurs in a subset of early stage disease led us to examine its potential as a marker for differentiating and subclassifying early stage CRCs.

### Correlation Between Maspin Expression and Clinicopathological Features

To explore correlations of maspin expression with clinicopathological features of colorectal cancer, we divided the cases into two groups with cytoplasmic IHC scores <150 (low) or ≥150 (high), respectively. Among the cohort of 628 cases with early stage cancers, 103 cases (16.4%) had high expression and 525 cases (83.6%) had low expression of cytoplasmic maspin. Using positive vs. negative nuclear staining as categories, 139 cases (22.1%) of the early stage cohort showed positive nuclear expression, whereas 489 cases (77.9%) showed no nuclear expression. Among the cohort of 115 cases with late stage cancers, 57.4% showed high cytoplasmic expression, and 22.6% showed positive nuclear expression of maspin.

In early stage CRCs (stages I and II; Table 1), the groups with high vs. low cytoplasmic maspin expression did not differ in terms of patient gender, patient age, histology, tumor location, tumor differentiation, or TNM stage. Early stage CRC groups stratified by nuclear expression of maspin did also not differ significantly in these parameters except for tumor differentiation, with nuclear positivity more frequently found in patients with poorly differentiated (G3) tumors. The mismatch repair status showed an association with both cytoplasmic and nuclear maspin expression. For the microsatellite instability (MSI) subtype, significantly higher rates of both high cytoplasmic and positive nuclear expression of maspin were observed compared to the microsatellite stable (MSS) subtype (Table 1). Among 145 MSI cases, 33 cases (22.8%) displayed high cytoplasmic expression, compared to only 14.5% (70/483) of MSS cases. Similarly, 31.7% of MSI cases but only 19.3% of MSS cases showed positive nuclear expression of maspin.

**Table 1:** Maspin expression and clinicopathological features of the early stage CRC cohort.

	**Maspin in cytoplasm (*****n*** **=** **628)**			**Maspin in nucleus (*****n*** **=** **628)**		
	**Low (*n* = 525)**	**High (*n* = 103)**	***p-*value**	**Negative (*n* = 489)**	**Positive (*n* = 139)**	***p*-value**
**Gender**			0.1957			0.2115
Male	274	46		256	64	
Female	251	57		233	75	
**Age**			0.1265			0.5593
≤ 65	228	36		209	55	
>65	297	67		280	84	
**Histology**			1.0000			0.1598
Mucinous	42	8		124	15	
Not mucinous	483	95		454	35	
**Tumor differentiation**			0.1665			0.0001
G1/2	486	91		461	116	
G3	39	12		28	23	
**Tumor location**			0.0526			0.3382
Left	270	42		248	64	
Right	255	61		241	75	
**Tumor stage**			0.1810			0.2331
I	187	44		186	45	
II	338	59		303	94	
**MMR status**			0.0215			0.0020
MSS	413	70		390	93	
MSI	112	33		99	46	

In 115 late stage colorectal cancer cases (stages III or IV), neither the high vs. low cytoplasmic expression groups nor the positive vs. negative nuclear expression groups differed significantly by patient gender, patient age, histology, tumor differentiation, or stage (Supplementary Table 2). Right-sided late stage CRCs showed higher nuclear (*p* = 0.0008) maspin expression than left-sided late stage cancers. Among the 103 late stage MSS cases, 12 (11.7%) and 20 (19.4%) cases showed high cytoplasmic and positive nuclear maspin expression, respectively. Among the 12 late stage cancer MSI cases, 2 (16.7%) showed high cytoplasmic expression and 6 (50.0%) cases showed positive nuclear expression. Late stage MSI cancers had a statistically significantly higher rate of positive nuclear maspin expression than MSS cancers (*p* = 0.0268).

### Correlation Between Maspin Expression and Patient Survival

**Figure 4:**
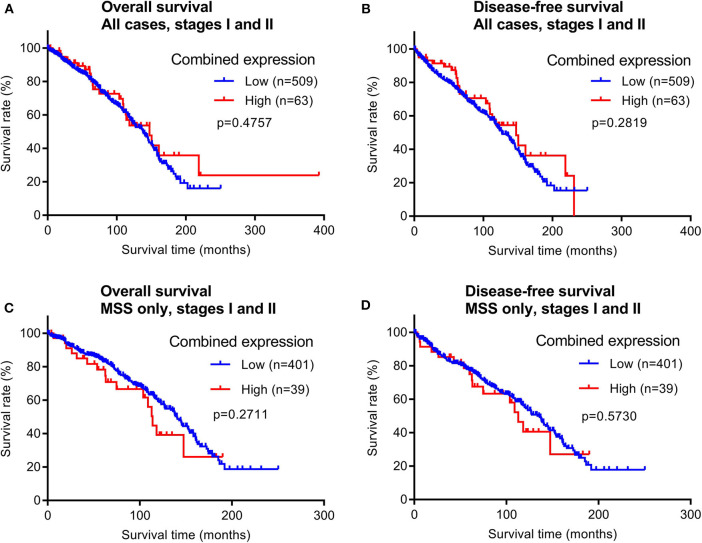
Overall survival and disease-free survival analyses of early stage CRCs (stages I and II) stratified by combined maspin protein expression (combined high category defined as cases with both high cytoplasmic and positive nuclear maspin protein expression). All other cases are defined as “low.” **(A,B)** All early stage CRCs, **(C,D)** early stage CRCs of only the MSS subtype.

To evaluate the prognostic potential of maspin protein expression for early stage CRCs, we examined the relationship between the patient survival time and maspin expression using Kaplan–Meier analyses. Of the 628 early stage cases examined by immunohistochemistry, 572 cases had recorded follow-up survival data with mean and median follow-up times of 80.2 and 71.1 months, respectively. These patients had not received adjuvant chemotherapy, which renders them a homogeneous and non-biased cohort that is ideal for prognostic relevance analyses. Both overall survival (OS) times and disease-free survival (DFS) times were examined for correlations with maspin expression levels. When either all early stage CRC cases combined or only the MSS subtype of early stage CRCs were considered, we did not observe any significant differences in either OS or DFS between groups with high vs. low expression of maspin (Figure 4). Median DFS for combined maspin expression was comparable at 62.6 months vs. 68.6 months for maspin-high vs. maspin-low early stage MSS patients.

**Figure 5:**
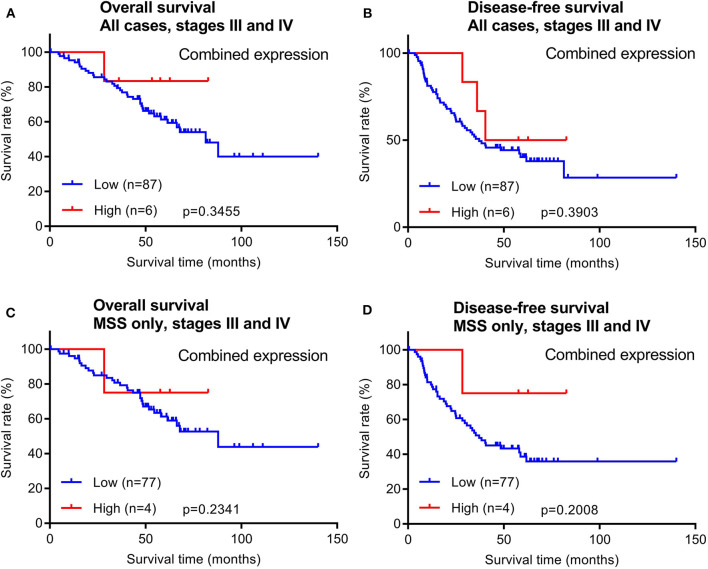
Overall survival and disease-free survival analyses of late stage CRCs (stages III and IV) stratified by combined maspin protein expression. **(A,B)** All late stage CRCs, **(C,D)** late stage CRCs of only the MSS.

In addition to early stage patients, we also evaluated 93 cases of late stage CRCs with available survival data. Similar to early stage patients, maspin protein levels did not stratify OS and DFS times in either all late stage CRC cases combined or in late stage MSS subtype CRCs (Figure 5).

**Figure 6:**
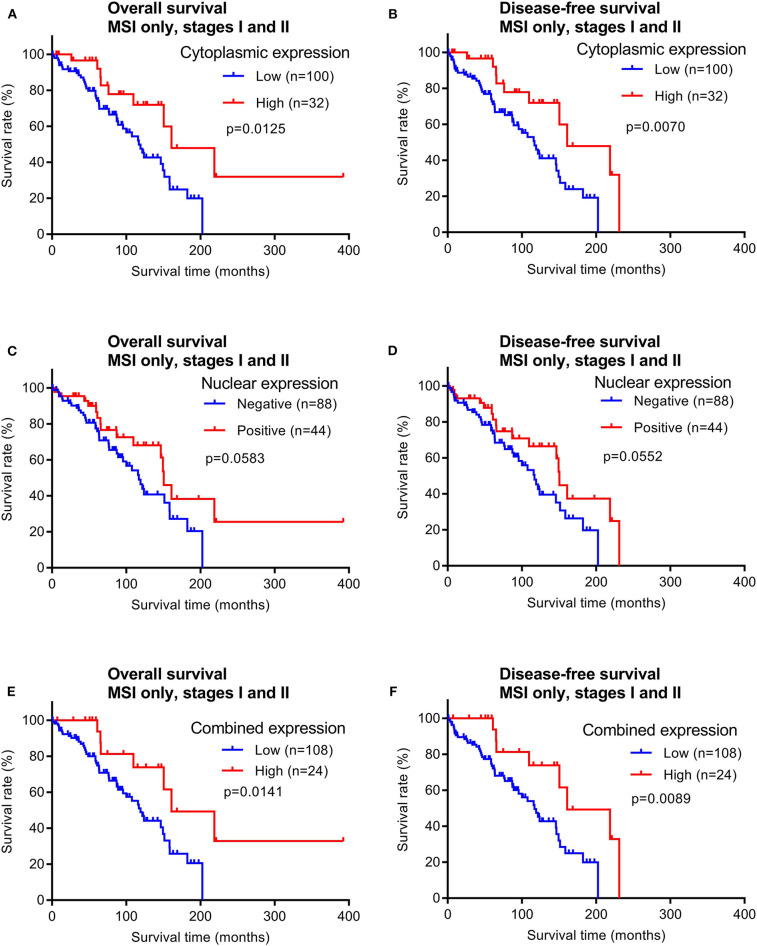
Overall survival and disease-free survival analyses of early stage MSI subtype CRCs (stages I and II) stratified by maspin protein expression. **(A,B)** Cytoplasmic expression, **(C,D)** nuclear expression, and **(E,F)** combined maspin expression.

In contrast, patients with early stage MSI subtype CRCs showed significant differences in both OS and DFS times between the groups with high vs. low cytoplasmic maspin expression (Figures 6A,B). Similar survival time trends were observed when only positive vs. negative nuclear expression was compared, although not statistically significant (Figures 6C,D). When high cytoplasmic and positive nuclear expression were considered as a combined high expression group [defined by high *H*-scores (≥150) and nuclear positivity], this group of early stage MSI cancer patients had significantly longer OS and DFS times (Figures 6E,F). Combined maspin-high patients had a median DFS of 85.5 vs. 62.7 months for combined maspin-low patients. There were only 12 late stage MSI CRC patients in our cohort, and thus examining correlations between survival times and maspin expression was not statistically feasible. The findings thus far indicated a potential of maspin overexpression as a favorable prognostic maker for early stage MSI CRC.

To test whether high maspin expression may serve as an independent prognostic marker for early stage MSI CRC, we performed univariate and multivariate analyses (Table 2). As expected, older patient age did significantly increase the hazards ratio for shorter survival. Other parameters, such as patient gender, tumor location, histology, tumor grade, and tumor stage were not predictive of early stage outcomes. However, these analyses did confirm that maspin protein overexpression was an independent predictor of favorable outcomes in patients with the MSI subtype of early stage CRC.

**Table 2:** Univariate and multivariate analyses of early stage MSI CRCs.

	**Overall survival**				**Disease-free survival**			
	Univariate^*^		Multivariate^*^		Univariate^*^		Multivariate^*^	
	**HR (95% CI)**	***p*-value**	**HR (95% CI)**	***p*-value**	**HR (95% CI)**	***p*-value**	**HR (95% CI)**	***p*-value**
Gender (male vs. female)	0.74 (0.41–1.28)	0.2861			0.76 (0.43–1.30)	0.3135		
Age (>65 vs. ≤ 65)	3.46 (1.83–7.11)	0.0001	3.27 (1.73–6.74)	0.0001	3.21 (1.74–6.39)	0.0001	3.04 (1.65–6.08)	0.0002
Tumor location (right vs. left)	1.71 (0.85–3.97)	0.1422			1.50 (0.76–3.31)	0.2550		
Histology (mucinous vs. others)	0.49 (0.17–1.12)	0.0975			0.46 (0.16–1.05)	0.0683		
Tumor grade (G3 vs. G1/2)	1.06 (0.53–1.98)	0.8509			1.11 (0.57–2.04)	0.7348		
Tumor stage (II vs. I)	1.46 (0.83–2.64)	0.1932			1.38 (0.80–2.48)	0.2464		
Maspin expression (high vs. low)	0.39 (0.17–0.79)	0.0076	0.43 (0.18–0.88)	0.0184	0.37 (0.16–0.74)	0.0038	0.39 (0.17–0.80)	0.0085

* *Cox proportional hazards model. HR, hazard ratio; CI, confidence interval*.

## Discussion

We used mass spectrometry proteomics to discover that maspin is overexpressed in a subset of CRCs. Using a validation cohort of 743 cases of CRCs across all clinical stages, we confirmed maspin protein overexpression in both cytoplasmic and nuclear compartments of CRCs. In particular, we found that maspin overexpression is correlated with better prognosis for the MSI subtype in early stage cancers (maspin-high mean OS of 110.9 months vs. maspin-low mean OS of 73.9 months), but not in the early stage MSS subtype (maspin-high mean OS of 70.7 months vs. maspin-low mean OS of 81.0 months) or late stage colorectal cancers. Our discovery was enabled by a large, well-annotated cohort of MSI and MSS patients with early stage colorectal cancer who had not received any adjuvant chemotherapy. Based on univariate and multivariate analyses, we then showed that maspin protein expression is an independent favorable prognostic marker for risk stratification of early stage MSI subtype CRC.

A uniform prognostic or predictive value of maspin protein expression in CRC has not been established in the literature. Several previous studies revealed no significant prognostic value for maspin. In a 156-case study of stage I/II colorectal cases, significant correlations were found between cytoplasmic expression and high tumor grade and between nuclear expression and tumor budding, but no difference between overall survival and maspin expression was found when the entire cohort was analyzed (16). In a 380-case study of stage II/III colorectal cancers randomized to adjuvant chemotherapy or to surgery only, maspin was found in most of the tumor cases with predominantly nuclear expression, and a significant treatment benefit was found in patients with low maspin expression but not in individuals with medium or high expression, although maspin expression levels were not significantly correlated with clinical outcomes in rectal cancer or control groups (17). In a 450-case study of advanced stage CRCs, nuclear and cytoplasmic maspin expression were assessed in both superficial and deep parts of the tumors (18). Among 13 clinicopathological features examined, the only association found was that right-sided tumors had stronger maspin expression and that maspin was not a significant prognostic factor (18). In a mixed cohort investigation, up-regulated maspin expression was involved in colorectal adenoma to adenocarcinoma progression but had no significant relationship with patient survival times (19). In another study of 243 stage II and 176 stage III colorectal cancers, maspin expression was differentially expressed in liver metastasis with early vs. prolonged time to recurrence after resection and was an independent predictor of time to recurrence and CRC-specific survival in stage III patients, but high expression did not correlate with survival in stage II patients (20).

Some studies have reported maspin expression as a prognostic marker of poor outcomes in CRC. In a 120-case study, high cytoplasmic expression was associated with a poor prognosis (21). In another 377-case study, maspin expression was reported to be a CEA-interacting marker, and strong maspin expression was associated with reduced disease-free and overall survival times (22). In a cohort of 172 patients with primary stage III colon cancer, nuclear maspin expression was found to be an independent adverse prognostic factor for overall survival and was highly predictive of adjuvant 5-FU chemotherapy response (23).

On the other hand, some studies have reported maspin as a marker of better prognosis in CRC. In a 121-case study, negative, cytoplasmic, nuclear, and mixed expression of maspin expression was observed in 9, 44, 24, and 23% of cases, respectively (24). The same study also reported that mixed cytoplasmic and nuclear expression of maspin was detected in 40% of MSI subtype cases and associated with better prognosis (24). In another study of 280 carcinomas and 80 adenomas, median recurrence-free survival was 80 months for maspin-positive cases vs. 42 months for maspin-negative cases, and the median overall survival was 98 months for maspin-positive patients vs. 57 months for maspin-negative patients (25).

Thus far, very few studies had addressed maspin in the MSI or MSS subtypes of colorectal cancer. One study analyzed 41 cases of MSI and 159 cases of MSS and found significant up-regulation of maspin in MSI colorectal tumors compared to MSS tumors or matched benign colonic mucosa (26). The same study also reported increased maspin expression in three MSI colon cancer cell lines (26). In a 216-case cohort MSI CRC of all stages, nuclear maspin expression was found in 51% of these cases and was molecularly associated with CIMP (CpG island methylator phenotype) rather than the MSI status (27). The same study also reported that positive nuclear maspin expression was associated with worse disease-free survival times (27). However, this conclusion was based on patients of all stages of CRC, and a large portion (124/216) of these patients had stage II and III cancers and had received adjuvant fluoropyridine-based therapy. Furthermore, the multivariate analysis failed to confirm a prognostic significance (27).

Based on published data, it is not straightforward to establish a generalized prognostic value for maspin across all types of CRC. In fact, we had hypothesized, and our results support the notion, that maspin plays a role in only a subset of CRCs, not all CRCs. We believe that this subtype-specific and early stage-restricted role of maspin is a major reason for the seemingly variable findings in the prior literature. Specifically, we show that maspin is a robust risk-stratifying marker only in early stage cancers of the MSI subtype. In many of the prior reports, study cohorts consisted of patients with heterogeneous cancer stage distributions, some of which had been treated with surgery alone, while others had also received adjuvant chemotherapy. In addition, results may vary due to differences in study designs and experimental methods, e.g., antibodies, immunochemical staining protocols, and scoring methods. Most importantly, cancer tissues are heterogeneous entities and careful selection of tissue cohorts with stringent criteria may be required to establish a role of maspin protein expression in colorectal cancer subtype risk stratification.

Future work is needed to elucidate the functional role of the enzyme maspin in MSI cancers and the mechanistic basis for maspin-associated differential clinical behavior. It is possible that maspin enhances immune recognition of MSI cancers (28, 29), and maspin might play a synergistic role during checkpoint inhibitor therapy. Our recent discovery that maspin is part of the CRC autoantigen-ome that elicits a cancer-directed humoral immune response is a promising step in this direction (7). In addition, it will be interesting to study whether patients with maspin-deficient early stage CRCs and expected poorer prognosis would benefit from more intense adjuvant therapies. Our study provides preliminary evidence that tissue protein expression of maspin is a prognostic marker for early stage MSI colorectal cancer. For eventual clinical application, further independent validation studies will be needed.

## Data Availability Statement

The datasets presented in this study can be found in online repositories. The names of the repository/repositories and accession number can be found below: ProteomeXchange (PXD019103).

## Ethics Statement

The study was approved by the Institutional Review Board of Memorial Sloan Kettering Cancer Center. Data were acquired retrospectively and in an anonymized manner such that patient consent was not required.

## Author Contributions

AT carried out most of the experiments, performed data analysis, and wrote a draft of the paper. YZ and MO assisted with experiments and data analysis. JS provided tissue resources and clinical annotation. DK provided partial funding and project advice. RH performed mass spectrometric experiments. JW and MR supervised the project, analyzed the data, and wrote the final manuscript. MR provided funding for the study.

## Conflict of Interest

JW is founder of and equity holder in Curandis. DK is a consultant for and equity holder in Paige and a consultant for Merck. MR is a member of the Scientific Advisory Boards of Trans-Hit, Proscia, and Universal DX. None of these companies had any influence in support, design, execution, data analysis, or any other aspect of this study. The remaining authors declare that the research was conducted in the absence of any commercial or financial relationships that could be construed as a potential conflict of interest.

## Abbreviations

CRC: colorectal cancer
IHC: immunohistochemistry
DFS: disease-free survival
OS overall survival.
